# PET/CT in the Staging and Treatment Response Assessment of Patients With Extranodal Marginal Zone Lymphoma

**DOI:** 10.1002/ajh.27712

**Published:** 2025-05-21

**Authors:** Juan Pablo Alderuccio, Russ A. Kuker, Eduardo Edelman Saul, Michele D. Stanchina, Mark K. Polar, Jennifer Chapman, Wei Zhao, Rafael Hennemann Sassi, Craig H. Moskowitz, Isildinha M. Reis, Izidore S. Lossos

**Affiliations:** ^1^ Division of Hematology, Department of Medicine, Sylvester Comprehensive Cancer Center University of Miami, Miller School of Medicine Miami Florida USA; ^2^ Division of Nuclear Medicine, Division of Radiology University of Miami, Miller School of Medicine Miami Florida USA; ^3^ Division of Hematopathology, Department of Pathology University of Miami, Miller School of Medicine Miami Florida USA; ^4^ Biostatistics and Bioinformatics Shared Resource Sylvester Comprehensive Cancer Center Miami Florida USA; ^5^ Department of Public Health Sciences University of Miami Miller School of Medicine Miami Florida USA

**Keywords:** extranodal marginal zone lymphoma, PET/CT, staging

## Abstract

^18^Fluorodeoxyglucose (FDG) positron emission tomography/computed tomography (PET/CT) is the standard imaging modality in lymphoma. The 2014 Lugano classification considers extranodal marginal zone lymphoma (EMZL) a non‐FDG‐avid disease, recommending contrast‐enhanced CT. We reassessed the utility of PET/CT for staging workup and response assessment of EMZL. We reviewed staging and response PET/CT of 190 EMZL sites from 152 patients. Each location was counted independently for patients with > 1 extranodal site. Although not standard, we considered FDG‐avid disease if SUVmax was ≥ 2, and calculated ratios between lymphoma SUVmax and mediastinal blood pool (BP index) and liver background (liver index). FDG avidity was detected in 151 (79.5%) out of 190 extranodal sites (in 117 [76.7%] out of 152 patients), with a median SUVmax of 4.5 (IQR 2.5–6.9, range 0–26.8). Locations showing FDG avidity in > 90% of extranodal sites included salivary gland, bone, lung, soft tissue, ocular adnexa, and airways. Skin was commonly non‐FDG avid (93.8%). Among 22 patients with > 1 extranodal location, there was concordant FDG avidity in all sites in 18 (81.8%) patients. Considering measurable extranodal disease size > 0.5 cm, we observed significant Pearson correlation coefficients (*r*) between lymphoma size and SUVmax (*r* = 0.20, *p* = 0.019, *n* = 142), BP index (*r* = 0.34, *p* < 0.001, *n* = 124), and liver index (*r* = 0.36, *p* < 0.001, *n* = 124). We also observed improved precision in response to treatment assessment in FDG‐avid EMZL tumors. This study demonstrates that EMZL is commonly an FDG‐avid disease, suggesting that PET/CT should be routinely used in the staging and response assessment workup of patients with EMZL.

## Graphical abstract

Extranodal marginal zone lymphoma (EMZL) is essentially an FDG‐avid disease. PET/CT improves the precision in the response assessment compared to contrast‐enhanced CT.

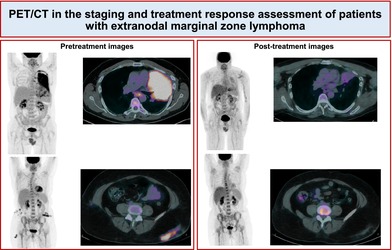

## 1 Introduction

^18^Fluorodeoxyglucose (FDG) positron emission tomography/computed tomography (PET/CT) possesses excellent sensitivity in detecting lymphoma and defining tumor burden. PET/CT is routinely implemented in the staging and treatment response assessment in practice and clinical trials during regulatory agencies' approval of novel agents [1]. Furthermore, accurate staging remains essential in defining a patient's prognosis and delineating the duration of therapy across many lymphoma subtypes [2, 3, 4, 5, 6]. The 2014 Lugano classification endorses PET/CT as the preferred imaging modality in lymphoma except for some indolent histologies, including marginal zone lymphoma (MZL). In patients with MZL, contrast‐enhanced computed tomography (CT) scans or magnetic resonance imaging (MRI) remain the preferred modalities, depending on disease location [7, 8]. In recent years, growing evidence has demonstrated FDG avidity in splenic and nodal MZL in more than 76% of the patients [9, 10, 11]. However, FDG avidity in extranodal marginal zone lymphoma (EMZL) remains controversial since it is influenced by disease location and tumor size and is suboptimal for detecting bone marrow involvement [12, 13, 14]. There is also limited data on the usage of PET‐CT for response assessment in EMZL.

MZL comprises 7% to 8% of all non‐Hodgkin lymphomas (NHL), with EMZL representing the most common subtype [15]. In pivotal clinical trials, EMZL has been frequently grouped with other MZL histologies and other indolent lymphomas, with results obtained for more common histologies such as follicular lymphoma applied to define the de facto standard‐of‐care for indolent lymphomas as a homogenous entity [16, 17]. However, EMZL is a disease characterized by a unique etiology, with antigen stimulation, genomic makeup, and clinical features that set it apart from other indolent lymphomas and MZL subtypes [18, 19]. EMZL may affect any tissue, with the most common locations including the stomach and ocular adnexa [15, 20]. FDG avidity in these locations has been reported in less than 50% of cases; however, other locations, such as the lung and head and neck extranodal structures, appear universally FDG‐avid [10, 13]. Nevertheless, lower FDG avidity could be related to small tumor size or associated with significant physiologic background activity rather than a true lack of tumor FDG uptake. Thus, we decided to determine the utility of PET/CT to detect EMZL across common disease locations during staging workup and response to treatment assessment in the modern era.

## 2 Methods

### 2.1 Patient Selection

We retrospectively analyzed the University of Miami MZL database, searching for patients with EMZL who underwent staging PET/CT. We found 152 patients diagnosed between February 2006 and January 2023 with PET/CT at diagnosis. Since 2019, the Sylvester Comprehensive Cancer Center at the University of Miami Lymphoma Program has consistently incorporated PET/CT in the staging workup of patients with EMZL independently of concerns for high‐grade transformation. Phillips and Siemens PET/CT scanners were used in this analysis. Images were reviewed by expert radiologists (R. A. K. and M. K. P.) using Thinking Systems software to ascertain FDG avidity of the tumor and exclude surrounding physiologic metabolic activity, with special attention to ocular adnexa and gastrointestinal locations. We defined gastrointestinal involvement as focal uptake above normal physiological activity. If scans were not available, we retrieved data from radiology reports (*n* = 15). Patients with excisional biopsy or another type of surgical resection that could affect PET/CT findings were excluded. In those patients with concomitant staging PET/CT and contrast‐enhanced CT, we determined the sensitivity and specificity of PET/CT to detect extranodal and nodal diseases and compared the two methods with respect to response to treatment. The University of Miami institutional review board approved this study.

Patients with high‐grade transformation at diagnosis were excluded. Patients with EMZL located in the adipose tissue, fascia, and skeletal muscle were designated as having soft tissue extranodal disease. We classified patients with lymphoma located in the sinuses, nasopharynx, oropharynx, larynx, and trachea as having airway extranodal disease. Each location was counted and analyzed independently for patients with more than one extranodal site. We considered lymphoma to be FDG‐avid if SUVmax was ≥ 2, as this SUVmax value is commonly observed between mediastinal blood pool (BP) and liver background values based on our accumulated experience with routine use of PET/CT in EMZL patients. When available, we also calculated ratios between lymphoma SUVmax and mediastinal blood pool (BP index), and between lymphoma SUVmax and liver background (liver index) in patients with measurable disease. Since smaller lesions are commonly observed in EMZL in vital organs and causing symptoms, we initially examined SUV activity across all the spectrum of reliably measured lesions with extranodal size starting from > 0.5 cm. Once we observed measurable SUV activity in lesions > 0.5 cm causing symptoms and confirmed by biopsies to be EMZL, we defined measurable disease as extranodal sizes starting from > 0.5 cm rather than > 1.0 cm as delineated in the Lugano Classification, since these frequently represent indications for treatment in EMZL.

### 2.2 Statistical Analyses

Categorical data were summarized using counts and percentages. Quantitative data were summarized using median, 1st and 3rd quartiles as the interquartile range (IQR), and minimum and maximum values as the range. Correlations between lymphoma size and SUVmax, BP index, and liver index were examined by Pearson correlation coefficients and scatterplots. The distribution of BP and liver indices by extranodal locations were depicted in boxplots. Statistical analyses were performed using SAS version 9.4 (SAS Institute Inc., Cary, North Carolina) and the r statistical software environment, version 3.4 (https://www.r‐project.org).

## 3 Results

### 3.1 FDG Avidity in the Staging of EMZL

The University of Miami MZL database had 580 patients with EMZL on July 7th, 2023, when data was collected. Thirteen patients with high‐grade transformation at diagnosis were excluded; 152 patients with staging PET/CT were included in this analysis. Among these 152 patients, 53 (34.8%) had concomitant staging contrast‐enhanced CT scans, and 35 at the time of treatment response (23%). Patient characteristics (Table S1) showed majorities for female sex (*n* = 88; 58%), Hispanic ethnicity (*n* = 77; 50.7%), normal lactate dehydrogenase (LDH, *n* = 126; 82.9%), and early‐stage disease (*n* = 98; 64.5%). The median age was 62 years (range 23–92). Most patients presented with a single extranodal site (*n* = 130, 85.5%), and 22 (14.5%) patients had more than one extranodal site (12 [7.9%], 4 [2.6%], and 6 [4%] patients with 2, 3, and 4 extranodal sites, respectively), providing a total of 190 locations. Among these 190 EMZL locations (Table 1), the most common sites were gastric (*n* = 33, 17.4%), ocular adnexa (*n* = 31, 16.3%), lung (*n* = 30, 15.8%), soft tissue (*n* = 19, 10%), skin (*n* = 16, 8.4%), and salivary gland (*n* = 13, 6.8%).

**TABLE 1:** FDG avidity by lymphoma location in a total 190 extranodal sites seen in *N* = 152 patients.

	Total		SUVmax < 2		SUVmax ≥ 2	
	*N*	Col%	*N*	Row%	*N*	Row%
Total EN sites	190	100	39	20.5	151	79.5
Location						
Ocular adnexa	31	16.3	2	6.5	29	93.5
Gastric	33	17.4	9	27.3	24	72.7
Lung	30	15.8	1	3.3	29	96.7
Soft tissue	19	10	1	5.3	18	94.7
Skin	16	8.4	15	93.8	1	6.3
Salivary gland	13	6.8	—	—	13	100
Airways	12	6.3	1	8.3	11	91.7
Breast	9	4.7	5	55.6	4	44.4
Colon	6	3.2	3	50	3	50
Bone	6	3.2	—	—	6	100
Liver	4	2.1	—	—	4	100
Non‐gastric GI	4	2.1	1	25	3	75
Tongue	2	1.1	—	—	2	100
Thyroid	2	1.1	—	—	2	100
Pancreas	1	0.5	1	100	—	—
Genitourinary	1	0.5	—	—	1	100
Adrenal gland	1	0.5	—	—	1	100
Ovary	—	—	—	—	—	—

Abbreviations: EN, extranodal; GI, gastrointestinal.

In all the 152 patients, the median SUVmax across sites was 4.5 (IQR 2.5–6.9; range 0–26.8, *n* = 190). Five patients had SUVmax > 14 (14.2, 19.6, 19.9, 25.9, and 26.8), all having biopsies from these areas ruling out transformation. Only one patient (SUVmax 19.9) presented with transformation 2 years and 6 months after diagnosis/initial PET/CT. In 106 patients, PET/CT detected 142 extranodal lesions that were measurable as per our definition (size > 0.5 cm). The remaining 48 extranodal lesions/sites were not measurable. In these 142 sites with measurable extranodal size, medians SUVmax and size were 5.5 (IQR 3.8–8.1; range 0–26.8) and 2.6 cm (IQR 1.8–3.7; range 0.7–18.9), respectively. BP and liver index data were available for 124 sites out of these 142 extranodal sites with measurable extranodal disease, with median BP and liver indexes of 3.72 (IQR 2.43–5.34; range 0–22.3) and 1.98 (IQR 1.34–2.83; range 0–9.24), respectively. If analyzed by the current Lugano criteria for PET positivity, FDG avidity above liver background at staging was observed in 89 (71.8%) patients.

### 3.2 Correlation Between FDG Avidity, Tumor Size and Location

**FIGURE 1:**
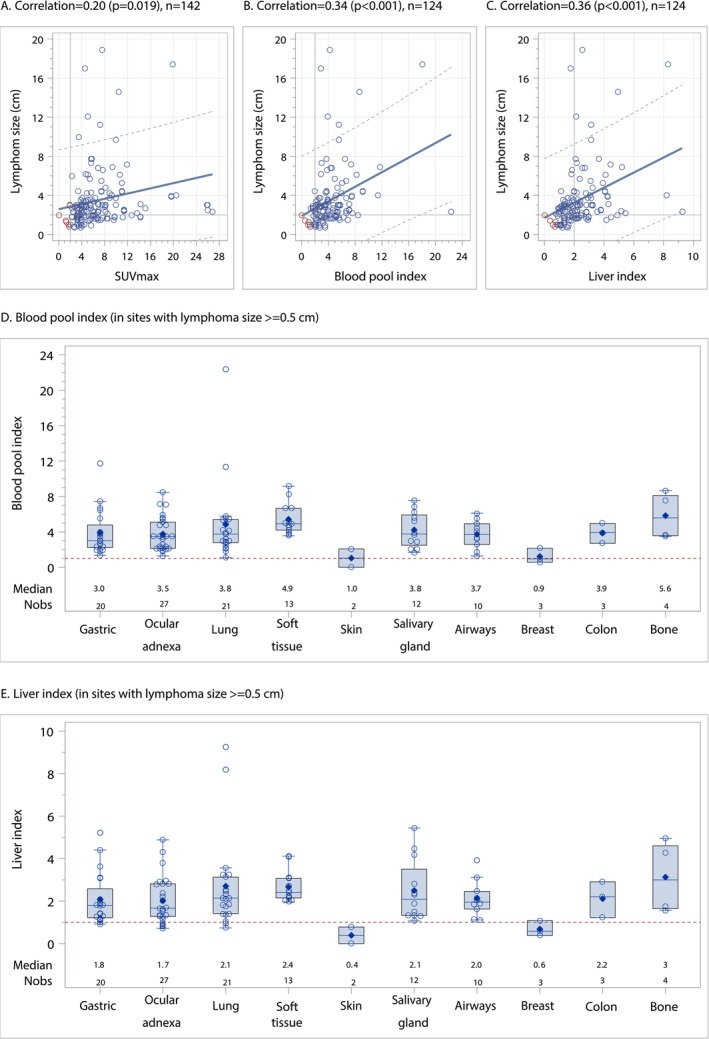
Characterization of PET avidity in EMZL. Pearson correlation coefficients between lymphoma size and SUVmax (A), blood pool index (B), and liver index (C) in sites with measurable disease (size > 0.5 cm). Red circles are sites with SUVmax < 2 (*n* = 6). Mediastinal blood pool (D) and liver (E) indexes in 10 selected extranodal sites with measurable disease (size > 0.5 cm). [Color figure can be viewed at wileyonlinelibrary.com]

Next, we examined the correlation between FDG avidity and tumor size in extranodal lesions with measurable disease (size > 0.5 cm). We observed statistically significant Pearson correlation coefficients (*r*) between lymphoma extranodal size and SUVmax (*r* = 0.20, *p* = 0.019, *n* = 142) (Figure 1A), BP index (*r* = 0.34, *p* < 0.001, *n* = 124) (Figure 1B), and liver index (*r* = 0.36, *p* < 0.001, *n* = 124) (Figure 1C). Including only measurable lesions with our proposed FDG‐avid disease definition (SUVmax ≥ 2), we observed similar significant correlations between lymphoma extranodal size and SUVmax (*r* = 0.17, *p* = 0.044, *n* = 136), BP index (*r* = 0.32; *p* < 0.001, *n* = 119), and liver index (*r* = 0.34, *p* < 0.001, *n* = 119) (Figure S1).

FDG avidity (SUVmax ≥ 2) was detected in 151 (79.5%) out of the 190 extranodal sites observed in 117 (77%) out of a total of 152 patients. Applying our proposed criteria of measurable extranodal size > 0.5 cm (*n* = 142 extranodal sites in 106 patients), FDG avidity (SUVmax ≥ 2) was detected in 136 (95.8%) sites seen in 103 patients (97.2%). Furthermore, FDG avidity (SUVmax ≥ 2) was present in 126 (96.9%) sites (in 95 patients [97.9%]) out of 130 EMZ sites (in 97 patients) which met the Lugano criteria of measurable size > 1 cm. Next, we evaluated SUVmax by tumor anatomical location, focusing on the most frequently involved sites (*n* > 4). SUVmax ≥ 2 was most observed in the salivary gland (100%), bone (100%), lung (96.7%), soft tissue (94.7%), ocular adnexa (93.5%), and airways (91.7%). In contrast, skin demonstrated the absence of FDG avidity (SUVmax < 2) in 15 (93.8%) of the 16 skin lesions (Table 1). Among 22 patients with more than one extranodal location/site, concordant FDG avidity was detected in 18 (81.8%) patients. SUVmax was ≥ 2 in 56 (93.3%) out of a total of 60 sites observed in these 22 patients (Table S2).

### 3.3 Comparing PET/CT Performance for Detecting Nodal and Extranodal Lesions With Contrast‐Enhanced CT

Next, we compared the detectability of extranodal lesions by PET/CT and current gold‐standard contrast‐enhanced CT scans. Among 70 positive extranodal sites on contrast‐enhanced CT scans, 63 (90%) were detected by PET‐CT, with only 1 of 63 (1.6%) being FDG non‐avid (SUVmax < 2). In terms of patients, of 53 patients with positive extranodal lesion(s) on contrast‐enhanced CT scans (gold standard), 46 (86.8%) patients also had positive lesions on PET/CT.

Among 47 patients with lymph node assessment by PET/CT and contrast‐enhanced CT scans, radiologically abnormal lymph nodes (> 1.5 cm and SUVmax > 2) by PET/CT were observed in 19 patients (median: 2.3 cm; range 1.6 to 17). Among them, 18 (94.7%) were avid by PET/CT (median SUVmax: 4.9; range 2.2 to 19). In only one case, lymph node involvement was detected by CT scan and not by PET/CT (size: 2.3 cm and SUVmax: 1.8). In contrast, 14 patients with lymph node size ≤ 1.5 cm (median: 1.3 cm, range 0.5 to 1.4 cm) demonstrated FDG avidity > 2 (median 4.1, range: 2.4 to 6.7), while 14 patients presented negative radiologic lymph nodes by contrast‐enhanced CT and PET/CT (median size: 1.2, range 0.5 to 1.5 cm; median SUVmax: 1.2, range 0 to 1.8). Compared to Lugano contrast‐enhanced CT scans (gold standard), the sensitivity, specificity, negative predictive value, and positive predictive value of PET/CT to detect nodal involvement were 94.7%, 53.6%, 93.8%, and 58.1%. The lower PET/CT specificity and positive predictive value compared to contrast‐enhanced CT might indicate MZL involvement of smaller lymph nodes, suggesting that PET/CT would lead to upstaging in EMZL.

For 95 patients, we had data on background FDG avidity for 124 measurable extranodal disease (size > 0.5 cm) sites. A total of 121 (97.6%) sites had a BP index ≥ 1, and 112 (90.3%) sites had a liver index ≥ 1 (Table 2). By location, the BP index was ≥ 1 in all cases except for extranodal lesions in breast (*n*/*N* = 1/3, 33%) and skin (*n*/*N* = 1/2, 50%) (Table 2, Figure 1D). Similarly, liver index ≥ 1 was observed in all cases except in gastric (*n*/*N* = 19/20, 95%), lung (*n*/*N* = 19/21, 90.5%), ocular adnexa (*n*/*N* = 22/27, 81.5%), and breast (*n*/*N* = 1/3, 33%). Furthermore, the two skin sites demonstrated a liver index < 1 (Table 2, Figure 1E). Thus, detection rates were 96% (*n* = 119), 97.6% (*n* = 121), and 90.3% (*n* = 112) by criteria of SUVmax ≥ 2, BP index ≥ 1, and liver index ≥ 1, respectively, underscoring similar results between indexes, especially between BP index and SUVmax (Table S3).

**TABLE 2:** Blood pool and liver indexes in 124 extranodal sites with measurable disease (size > 0.5 cm).

Variable	Total		Blood pool index (Site SUVmax/blood pool activity)						Blood pool index				Liver index (Site SUVmax over background liver activity)					Liver index			
									≥ 1		< 1						
	≥ 1		< 1	
*N*	Col%	*N*	Mean	SD	Median	Min	Max	*N*	Row%	*N*	Row%	Mean	SD	Median	Min	Max	*N*	Row%	*N*	Row%
Total	124	100.0	124	4.29	2.94	3.72	0.00	22.33	121	97.6	3	2.4	2.32	1.49	1.98	0.00	9.24	112	90.3	12	9.7
EN site/location																					
Ocular adnexa	27	21.8	27	3.76	1.88	3.48	1.29	8.47	27	100.0	—	—	2.02	1.09	1.67	0.72	4.88	22	81.5	5	18.5
Lung	21	16.9	21	4.82	4.56	3.75	1.08	22.33	21	100.0	—	—	2.70	2.15	2.14	0.74	9.24	19	90.5	2	9.5
Gastric	20	16.1	20	3.92	2.54	3.00	1.35	11.75	20	100.0	—	—	2.08	1.20	1.80	0.92	5.22	19	95.0	1	5.0
Soft tissue	13	10.5	13	5.43	1.75	4.92	3.56	9.17	13	100.0	—	—	2.67	0.73	2.40	1.97	4.12	13	100.0	—	—
Salivary gland	12	9.7	12	4.23	2.01	3.77	1.68	7.56	12	100.0	—	—	2.50	1.45	2.09	1.07	5.44	12	100.0	—	—
Airways	10	8.1	10	3.73	1.59	3.72	1.29	6.11	10	100.0	—	—	2.13	0.87	1.95	1.12	3.93	10	100.0	—	—
Bone	4	3.2	4	5.84	2.66	5.59	3.50	8.67	4	100.0	—	—	3.13	1.73	3.00	1.56	4.95	4	100.0	—	—
Liver	4	3.2	4	4.01	1.13	4.10	2.56	5.29	4	100.0	—	—	2.12	0.41	2.18	1.58	2.56	4	100.0	—	—
Breast	3	2.4	3	1.22	0.83	0.94	0.57	2.16	1	33.3	2	66.7	0.68	0.36	0.59	0.39	1.08	1	33.3	2	66.7
Colon	3	2.4	3	3.86	1.12	3.93	2.71	4.95	3	100.0	—	—	2.11	0.85	2.20	1.23	2.91	3	100.0	—	—
Skin	2	1.6	2	1.04	1.47	1.04	0.00	2.07	1	50.0	1	50.0	0.39	0.55	0.39	0.00	0.78	—	—	2	100.0
Adrenal gland	1	0.8	1	2.61	—	2.61	2.61	2.61	1	100.0	—	—	1.57	—	1.57	1.57	1.57	1	100.0	—	—
Non—gastric GI	1	0.8	1	18.09	—	18.09	18.09	18.09	1	100.0	—	—	8.29	—	8.29	8.29	8.29	1	100.0	—	—
Genitourinary	1	0.8	1	3.92	—	3.92	3.92	3.92	1	100.0	—	—	1.96	—	1.96	1.96	1.96	1	100.0	—	—
Thyroid	1	0.8	1	3.82	—	3.82	3.82	3.82	1	100.0	—	—	1.75	—	1.75	1.75	1.75	1	100.0	—	—
Tongue	1	0.8	1	6.71	—	6.71	6.71	6.71	1	100.0	—	—	5.18	—	5.18	5.18	5.18	1	100.0	—	—

*Note:* EN sites/locations listed by descending order of total *n*.

Abbreviations: EN, extranodal; GI, gastrointestinal; max, maximum; min, minimum; SD, standard deviation.

### 3.4 PET/CT in the Response Assessment of EMZL

Next, we evaluated the applicability of PET/CT in assessing response in EMZL patients. A total of 79 patients with 104 lymphoma sites underwent end‐of‐treatment PET/CT with the following patient‐level responses available for 78 patients: CR in 60 (76.9%), PR in 9 (11.5%), and PD in 9 (11.5%).

**FIGURE 2:**
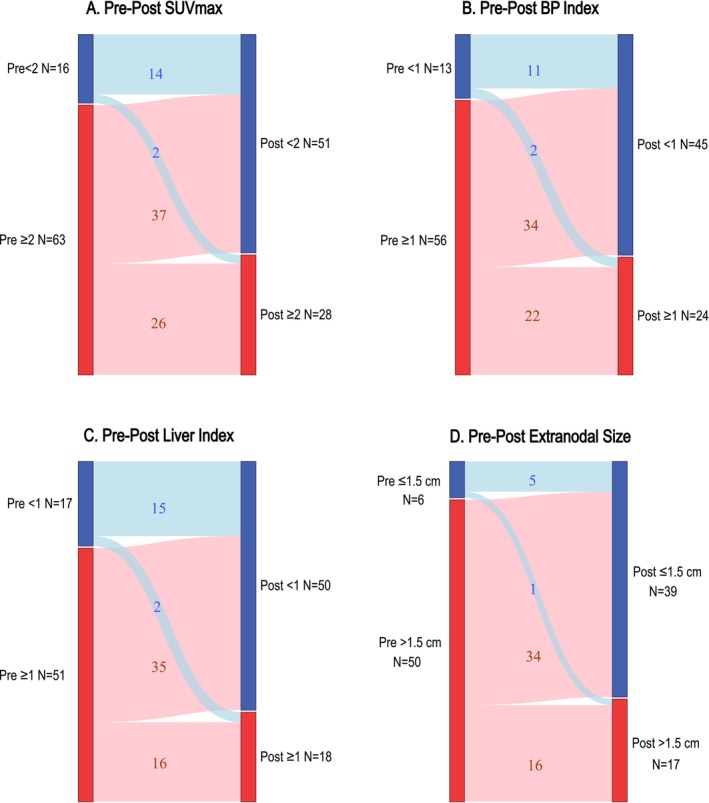
Changes in pre and post‐treatment in lymphoma SUVmax (A), blood pool index (B), and liver index (C), and extranodal size (D) in patients with measurable disease. [Color figure can be viewed at wileyonlinelibrary.com]

We then analyzed changes in SUVmax, BP, and liver indexes, and tumor size from pre‐to‐post‐treatment studies. Changes in SUVmax following treatment were analyzed in 79 patients (one extranodal site per patient) (Figure 2A). In 16 patients with pre‐treatment SUVmax < 2, the post SUVmax remained in the same range of < 2 in 14 (87.5%), and post SUVmax became ≥ 2 in 2 (12.5%) patients. In 63 patients with pre‐treatment SUVmax ≥ 2, there was a response (defined as post SUVmax < 2) in 37 (58.7%) patients and no response (post SUVmax ≥ 2) in 26 (41.3%) patients.

Most patients with a pre‐treatment BP index ≥ 1 (*n* = 56) decreased to < 1 (*n* = 34, 60.7%) in post‐treatment PET/CT. The pre‐treatment BP index increased from < 1 to ≥ 1 in post‐treatment PET/CT in only two patients (Figure 2B). Similarly, of the *n* = 51 with pre‐treatment liver index ≥ 1, 35 (68.6%) patients demonstrated a post‐treatment liver index < 1, and 16 (31.4%) had persistent post‐treatment liver index ≥ 1 (Figure 2C). The same two patients demonstrated post‐treatment indexes ≥ 1 compared to pre‐treatment < 1, compatible with disease progression.

To compare response assessment by PET/CT avidity and extranodal site measurement, we examined images from 56 patients with pre‐ and post‐treatment data, including 50 (89.3%) patients with pre‐treatment lesions > 1.5 cm, a size limit used in Lugano criteria to define abnormal lymph node size, and 6 (10.7%) patients with size ≤ 1.5 cm (Figure 2D). In the latter six patients, pre‐treatment SUVmax was ≥ 2 in five patients and < 2 in one (SUVmax = 1.2). In 5 (83.3%) of these six patients, post‐treatment size remained ≤ 1.5 cm, and in one increased to > 1.5 cm (size = 2.1 cm) with SUVmax > 2 (SUVmax = 3.8). In the 50 patients with pre‐treatment lesions > 1.5 cm, pre‐treatment SUVmax was ≥ 2 in all these patients. After treatment, in 34 (68%) patients, the size decreased to ≤ 1.5 cm with SUVmax ≥ 2 in six of these patients and < 2 in 27 (and unknown SUVmax for one patient), while in the remaining 16 (32%) patients with post‐treatment size > 1.5 cm, 14 had SUVmax ≥ 2 and *N* = 2 had SUVmax < 2.

Finally, 35 patients had concomitant end‐of‐treatment PET/CT and contrast‐enhanced CT. 27 (77.1%) patients achieved CR by PET/CT criteria, including 19 (54.2%) patients who achieved CR by contrast‐enhanced CT and eight (22.8%) patients still presented measurable disease on contrast‐enhanced CT (range 0.3 to 5.3 cm), with three patients being > 1.5 cm (2.1 to 5.3 cm).

## 4 Discussion

This study represents the largest analysis evaluating the avidity and applicability of PET/CT for staging and response assessment in EMZL. Furthermore, in contrast to most previous publications, in this study, images were re‐reviewed and reassessed, and each lesion was re‐assessed individually. We demonstrated that EMZL is essentially an FDG‐avid disease in more than 76% of the cases, with nearly universal uptake at specific locations such as the salivary gland, airways, lung, soft tissue, bone, and liver. Conversely, skin was commonly not FDG avid, underscoring the need for other methodologies of disease assessment like physical examination and photography. This finding aligns with the proposed reclassification of this entity in the 5th Edition of the World Health Organization Classification of Lymphoid Tumors and International Consensus Classification of Mature Lymphoid Neoplasms as primary cutaneous marginal zone lymphoproliferative disorder due to distinctly indolent clinical behavior [21, 22]. Interestingly, we observed that patients with more than one extranodal location demonstrated congruent FDG‐avidity across all sites, except when the skin was involved, underscoring the value of PET/CT to stage and detect disseminated disease precisely. Compared to other indolent NHL, such as follicular lymphoma, staging PET/CT demonstrates FDG avidity in a higher proportion of these patients, approximately 98%, including extranodal locations [23, 24].

The heterogeneity in EMZL presentation and lower incidence of nodal involvement may explain the variability in FDG uptake observed and highlight the need for analyses of large cohorts to determine the value of PET/CT in different locations [20]. Prior studies included a small number of patients; however, more extensive studies (with *n* > 70) described FDG avidity in between 31.8% and 75% of EMZL cases [25, 26, 27]. These studies also evaluated FDG avidity by lymphoma location; however, they were relatively small to provide definitive conclusions. Variability by disease location appears to be the main limiting factor to regularly incorporating PET/CT in the workup of these patients. Overall, FDG avidity has been previously reported as follows: soft tissue (70% to 100%), lung (50% to 100%), head and neck (50% to 100%), ocular adnexa (22% to 71%), stomach (20% to 52%), breast (33%), and skin (10% to 23%) [25, 26, 28, 29]. In the present study, we observed similar increased FDG avidity in the salivary gland (100%), lung (96.7%), soft tissue (94.7%), and airways (91.7%) aligned with prior observations. In contrast, skin had low rates of FDG avidity (6.3%). However, we observed more variability compared to prior reports in ocular adnexa (93.5%), gastric (72.7%), and breast (44.4%) locations. Furthermore, all patients in our study underwent PET/CT independently of perceived disease aggressiveness, decreasing selection bias towards patients with possible high‐grade transformation or disseminated lymphoma.

We also attempted to identify which SUVmax threshold best informs tumor identification in patients with EMZL. Focal uptake usually discriminates tumor recognition from background physiological activity. We also hypothesized that the lack of FDG uptake is secondary to small size rather than a lack of avidity in many cases. To this end, we observed a significant correlation between lymphoma size and FDG avidity. To avoid variability in SUVmax values commonly observed secondary to PET scanner and FDG administration protocol, we compared SUVmax with BP and liver background activity and developed corresponding indexes. In the index analyses, we also observed a significant correlation across locations when we included patients with measurable disease; this correlation was not observed for skin and breast locations, but the number of patients included with those sites was small. Pretreatment BP and liver index were similar to SUVmax, but indexes are normalized to background activity, providing internal control for each patient. The rationale for using different criteria to identify PET‐avid extranodal lesions in EMZL in pretreatment staging scans from those stipulated by the 2014 Lugano Classification is our experience observing an SUVmax ≥ 2 in the lymphoma area and a size of < 1.0 cm in staging PET/CT. Furthermore, we also observed a more precise response assessment by PET/CT compared to contrast‐enhanced CT. A significant number of patients (22.8%) achieved CR by PET/CT criteria but still presented measurable disease by CT criteria, underscoring relevant inconsistencies impacting practice and clinical trials. For assessment of response, we used a posttreatment decrease of the extranodal lesion to < 1.5 cm as an acceptable cutoff, as residual lesions without FDG avidity and negative biopsies are commonly seen in patients with initially large EMZL lesions and are similar to the cutoff suggested in the Lugano classification for normal‐size lymph nodes. Response criteria for extranodal lesions in indolent lymphomas were not specifically defined in the 2014 Lugano Classification, and complete disappearance of extranodal lesions suggested as a criterion for CR in the Lugano Classification is not applicable for EMZL, underscoring the need for studies providing guidance in these patients.

Our study represents the largest‐ever analysis of PET/CT in EMZL, indicating that most patients have FDG‐avid disease. This finding suggests that PET/CT should be the staging and response assessment methodology of choice in EMZL, as it is for many other lymphomas. The thresholds used for the definition of avidity need to be adjusted for EMZL compared to other NHLs, as we demonstrate herein. We strongly suggest that in patients with FDG‐avid EMZL, this imaging modality can be used to assess treatment response more accurately. In our extensive institutional experience (Figure S2), we have repeatedly observed the resolution of focal SUVmax to values below BP in patients responding to treatment. Patients with non‐avid EMZL, as determined by initial PET/CT, should be followed with contrast‐enhanced CT alone or MRI, as is currently standard for EMZL patients. We have already incorporated this approach in MZL‐specific clinical trials (NCT05296070, NCT06569680, and NCT06796998), in which patients undergo PET/CT and contrast‐enhanced CT/MRIs at screening. Those with FDG‐avid disease are assessed for response using PET/CT, while patients with non‐FDG‐avid disease are followed by contrast‐enhanced CTs or MRI, depending on disease location.

In conclusion, PET/CT should be included in the staging and response assessment of patients with EMZL, acknowledging possible limited sensitivity in skin and gastrointestinal locations, the latter due to physiological uptake. Based on our results, we advocate for PET/CT inclusion as the standard imaging modality in EMZL in the planned revision of Lugano classification guidelines, as discussed during the 2023 International Conference on Malignant Lymphoma workshop. Future studies should assess the appropriate threshold for response assessment. However, a decreased SUVmax threshold of < 2 or comparison to mediastinal BP or background of the organ involved rather than liver background activity should be considered reasonable alternatives. In future prospective studies, it would be beneficial to examine the comparison of tumor avidity to the background of the organ involved as a potentially another method to assess response to treatment in extranodal locations.

## Author Contributions

Juan Pablo Alderuccio and Izidore S. Lossos designed and performed the research, analyzed the data, were involved in data acquisition, and wrote the manuscript with input and approval of the final version from all coauthors; Isildinha M. Reis and Wei Zhao contributed to the statistical analysis and critically reviewed the manuscript; Russ A. Kuker, Eduardo Edelman Saul, Michele D. Stanchina, Mark K. Polar, Jennifer Chapman, Wei Zhao, Rafael Hennemann Sassi, Craig H. Moskowitz, and Isildinha M. Reis designed and performed the research, analyzed the data, were involved in data acquisition, and critically reviewed and approved the manuscript. All authors approved this manuscript for publication.

## Conflicts of Interest

Juan Pablo Alderuccio: Research support: ADC Therapeutics, Genmab, AbbVie, and BeiGene. Consultancy: ADC Therapeutics, Genentech, Genmab, AbbVie, Novartis, Lilly, and Regeneron. Izidore S. Lossos: Compensation for teaching: ADC Therapeutics: Consultancy: Adaptive Biotechnology; Research support: ADC Therapeutics, Genentech. The other authors declare no conflicts of interest.

## Supporting information


**DATA S1.** Supporting Information.

## Data Availability

Qualified investigators may request de‐identified data from this study by contacting the corresponding author.
