# Supplementary material for: PET/CT in the Staging and Treatment Response Assessment of Patients With Extranodal Marginal Zone Lymphoma
Source: Am J Hematol. 2025 May 21;100(8):1295–304. doi: 10.1002/ajh.27712 (PMC12232532; doi:10.1002/ajh.27712)
Supplement: Supplementary file 1 — DATA S1. Supporting Information. [file AJH-100-1295-s001.docx]

| **Supplemental Table 1**. Baseline characteristics | | |
| --- | --- | --- |
|  | **N** | **%** |
| **Total** | 152 | 100.0 |
| **Sex** |  |  |
| Female | 88 | 57.9 |
| Male | 64 | 42.1 |
| **Ethnicity** |  |  |
| Non-Hispanic | 53 | 34.9 |
| Hispanic | 77 | 50.7 |
| Unknown | 22 | 14.5 |
| **Race/Ethnicity** |  |  |
| Non-Hispanic White | 45 | 29.6 |
| Hispanic White | 42 | 27.6 |
| Black | 10 | 6.6 |
| Other | 36 | 23.7 |
| Unknown | 19 | 12.5 |
| **LDH** |  |  |
| Normal LDH | 126 | 82.9 |
| Elevated LDH | 18 | 11.8 |
| Unknown | 8 | 5.3 |
| **Stage** |  |  |
| Stage I-II | 98 | 64.5 |
| Stage III-IV | 54 | 35.5 |
| Unknown | -- | -- |
| **No. of Extranodal sites** |  |  |
| 1 | 130 | 85 5 |
| 2 | 12 | 7.9 |
| 3 | 4 | 2.6 |
| 4 | 6 | 4.0 |
| 5 | -- | -- |
| Abbreviations: LDH, lactate dehydrogenase. | | |

| **Supplemental Table 2.** SUVmax and lymphoma size (cm) in 22 patients with multiple extranodal (EN) sites  (Total 60 sites in 12, 4, and 6 patients with 2, 3, and 4 EN sites, respectively.) | | | | | | | | | | | | | |
| --- | --- | --- | --- | --- | --- | --- | --- | --- | --- | --- | --- | --- | --- |
| **Row** | **No of**  **sites** | **EN 1** | **EN 1**  **SUVmax** | **EN 1**  **Size** | **EN 2** | **EN 2**  **SUVmax** | **EN 2**  **Size** | **EN 3** | **EN 3**  **SUVmax** | **EN 3**  **Size** | **EN 4** | **EN 4**  **SUVmax** | **EN 4**  **Size** |
| 1 | 2 | lung | 8.9 | 3.7 | skin | **0** | **2** |  |  |  |  |  |  |
| 2 | 2 | lung | 8.1 | 3 | ocular adnexa | **1.8** | **0.8** |  |  |  |  |  |  |
| 3 | 2 | gastric | 11.9 | 7.2 | liver | 6.9 | 6.8 |  |  |  |  |  |  |
| 4 | 2 | gastric | 9.4 | 6.9 | bone | 2.8 | 2.8 |  |  |  |  |  |  |
| 5 | 2 | lung | 4.9 | 4.2 | soft tissue | 3.5 | 10 |  |  |  |  |  |  |
| 6 | 2 | airways | 7.7 | 1.7 | ocular adnexa | 8 | 2.8 |  |  |  |  |  |  |
| 7 | 2 | liver | 3.7 | 2 | soft tissue | 3.5 | 1.9 |  |  |  |  |  |  |
| 8 | 2 | Ocular adnexa | 6.8 | 1.5 | soft tissue | 4.5 | 2 |  |  |  |  |  |  |
| 9 | 2 | soft tissue | 10 | 3.7 | ocular adnexa | 8.9 | 4.2 |  |  |  |  |  |  |
| 10 | 2 | salivary gland | 6.8 | 2.3 | lung | 5.1 | 12.1 |  |  |  |  |  |  |
| 11 | 2 | ocular adnexa | 5.4 | 1.5 | soft tissue | **1.9** | **3** |  |  |  |  |  |  |
| 12 | 2 | salivary gland | 6 | 1.9 | ocular adnexa | 3.8 | 1 |  |  |  |  |  |  |
| 13 | 3 | salivary gland | 13.6 | 2.2 | salivary gland | 11.2 | 1.8 | lung | 20.5 | 4 |  |  |  |
| 14 | 3 | soft tissue | 9.2 | 4 | lung | 7.6 | 18.9 | adrenal gland | 4.7 | 2.8 |  |  |  |
| 15 | 3 | gastric | 7.4 | 5.5 | airway | 4.5 | 2 | salivary gland | 3.2 | 1.8 |  |  |  |
| 16 | 3 | lung | 5.1 | 6.1 | soft tissue | 2.3 | 6 | gastric | 4.7 | - |  |  |  |
| 17 | 4 | bone | 25.9 | 3 | soft tissue | 26 | 2.5 | gastric | 14.2 | 1.8 | bone | 15.1 | 2.7 |
| 18 | 4 | lung | 26.8 | 2.3 | salivary gland | 6.7 | 1.7 | bone | 5.9 | 2.4 | bone | 5.7 | 2.7 |
| 19 | 4 | lung | 6.2 | 2 | soft tissue | 5.6 | 1.6 | soft tissue | 6 | 3.4 | soft tissue | 6.8 | 6.8 |
| 20 | 4 | gastric | 10 | 3 | lung | 3.3 | 1 | liver | 7 | 2.5 | soft tissue | 10 | 9.7 |
| 21 | 4 | soft tissue | 5.7 | 4.3 | lung | 4.8 | 2.9 | liver | 4.1 | 3.7 | gastric | 4.7 | 6.2 |
| 22 | 4 | salivary gland | 4.3 | 3.4 | lung | 5.4 | 5.7 | bone | 4 | 3 | colon | **1.5** | **0** |
| EN, extranodal; Bold: four sites with SUVmax <2 (Rows 1, 2, 11 & 22).  * Not shown, data for 130 (85.5%) out of 152 patients with a single EN site. | | | | | | | | | | | | | |

| **Supplemental Table 3.** Sites identified using SUVmax 2.0 or blood pool ≥1 rather than liver index ≥1  among n=124 extranodal sites with measurable size >0.5cm | | | | | |
| --- | --- | --- | --- | --- | --- |
| **SUVmax** | **Blood pool index** | **Liver index** | **Frequency** | **Percent** | **Cumulative Frequency** |
| ≥2 | ≥1 | ≥1 | 112 | 90.3 | 112 |
| ≥2 | ≥1 | <1 | 7 | 5.7 | 119 |
| <2 | ≥1 | <1 | 2 | 1.6 | 121 |
| <2 | <1 | <1 | 3 | 2.4 | 124 |
| The detection rates were 96.0% (n=119), 97.6% (n=121), and 90.3% (n=112) by SUVmax, BP index, and liver index, respectively. | | | | | |


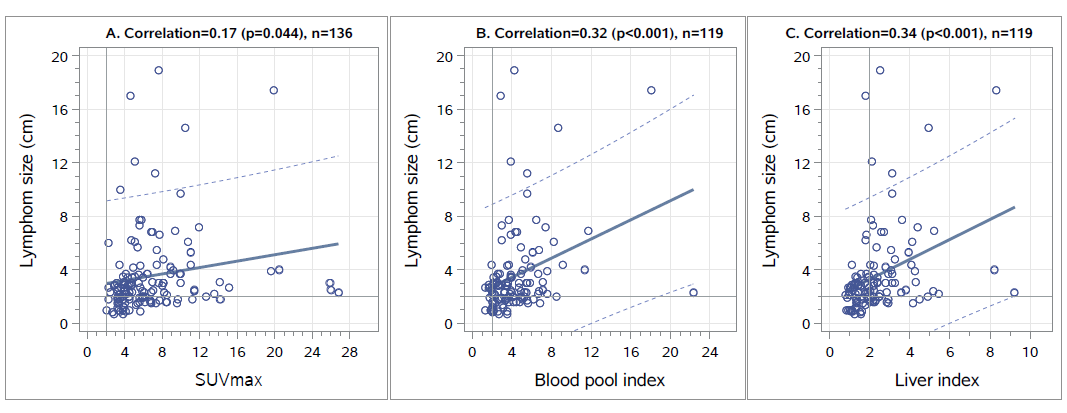


**Supplemental Figure 1.** Correlation between lymphoma size and SUVmax (A), blood pool index (B), and liver index (C) in sites with measurable disease (size >0.5 cm) and SUVmax >2).

**
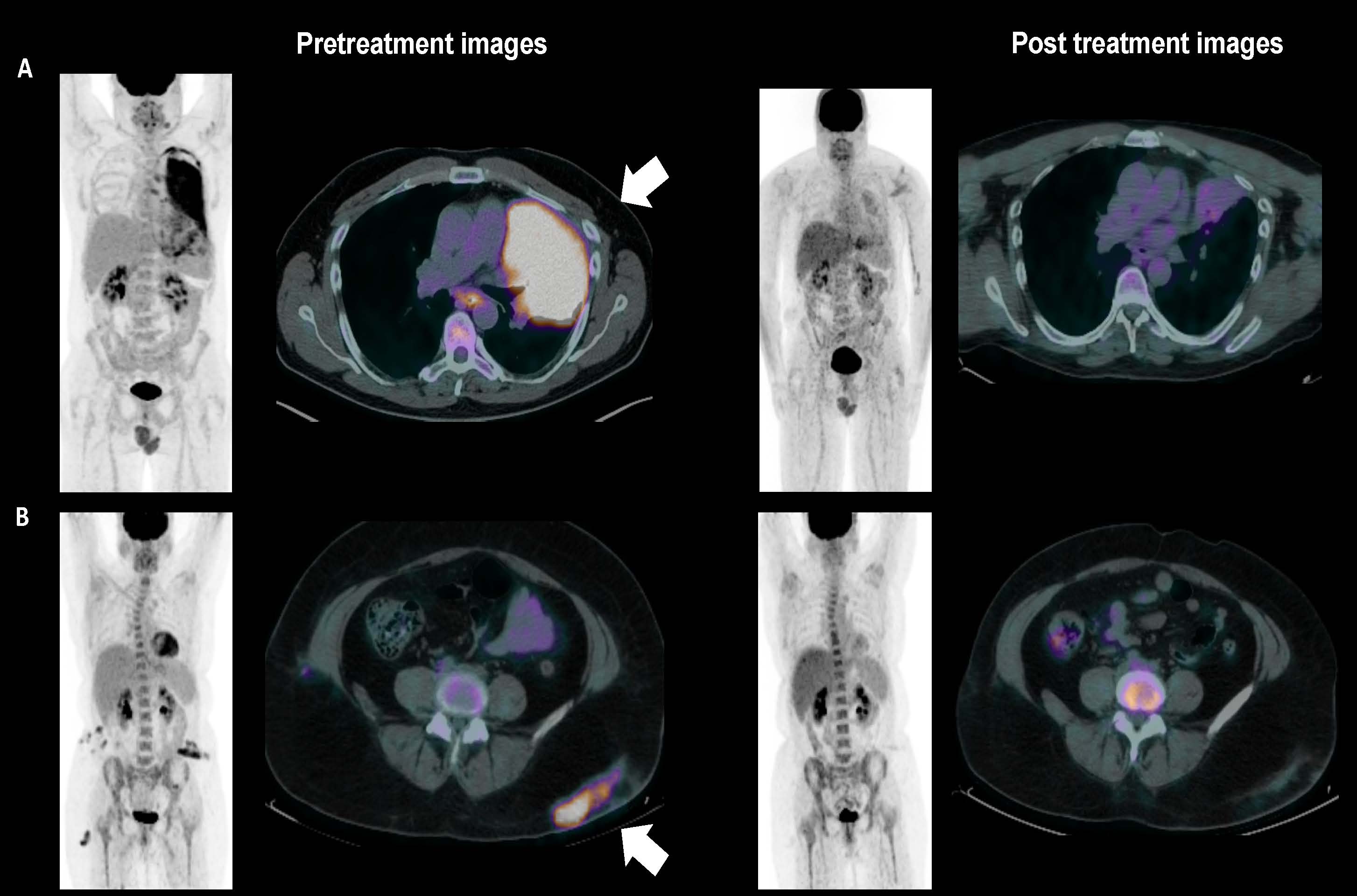
**

**Supplemental Figure 2.** FDG-avid lesions in staging PET/CT and response assessment in patients with extranodal marginal zone lymphoma. (**A**) Lung and (**B**) soft tissue.
